# Natural variation in cold tolerance in the nematode *Pristionchus pacificus*: the role of genotype and environment

**DOI:** 10.1242/bio.20148888

**Published:** 2014-08-22

**Authors:** Angela McGaughran, Ralf J. Sommer

**Affiliations:** Department for Integrative Evolutionary Biology, Max Planck Institute for Developmental Biology, 72076 Tübingen, Germany; ‡ Present address: CSIRO Land and Water, Black Mountain Laboratories, Clunies Ross Street, Canberra ACT 2601, Australia.; § Present address: University of Melbourne, Department of Genetics/Bio21 Institute, 30 Flemington Road, Melbourne VIC 3031, Australia.

**Keywords:** Cold tolerance, Genotype-by-environment interaction, Nematode, Plasticity, *Pristionchus pacificus*

## Abstract

Low temperature is a primary determinant of growth and survival among organisms and almost all animals need to withstand temperature fluctuations in their surroundings. We used the hermaphroditic nematode *Pristionchus pacificus* to examine variation in cold tolerance in samples collected from 18 widespread locations. Samples were challenged by exposure to both direct and gradual low temperature after culture in the laboratory at 20°C. A short-term acclimation treatment was also applied to assess cold tolerance following a pre-exposure cold treatment. Finally, genotype-by-environment (G × E) analysis was performed on a subset of samples cultured at two additional temperatures (15°C and 25°C). *P. pacificus* displayed a high degree of natural variation in cold tolerance, corresponding to the presence of three distinct phenotypic classes among samples: cold tolerant, non-cold tolerant, cold tolerant plastic. Survival of gradual cold exposure was significantly higher than survival of direct exposure to low temperature and a cold exposure pre-treatment significantly enhanced cold tolerance in some samples. By focusing on a sub-set of well-sampled locations from tropical La Réunion Island, we found evidence of significant effects of genotype and environment on cold tolerance, and we also showed that, within the different Réunion locations sampled, all three phenotypic classes are generally well represented. Taken together, our results show that *P. pacificus* exhibits a highly plastic tolerance to cold exposure that may be partly driven by differential trait sensitivity in diverse environments.

## INTRODUCTION

Throughout the diversity of life, all organisms are constrained by their physiological ability to respond to environmental variability at multiple temporal and spatial scales. This manifests in the physical distribution of species being defined by their ability to withstand the stresses imposed by their local environment (e.g. Hoffmann and Parsons, 1991). Determining the factors that aid organism survival of environmental stress exposure is important for our understanding of physiology, ecology and evolution. Further, evaluating the extent of variation in physiological response across environments is necessary for characterisation of evolutionary processes (Cutter et al., 2010).

From a phenotypic perspective, many organisms have been shown to cope with variable environments by exhibiting plasticity. Plastic responses refer to the change in expressed phenotype of a given genotype as a function of the environment (e.g. Scheiner, 1993) and broadly include physiological reactions to environmental factors (Wharton, 2011). Employing plasticity in varied environments may maximise fitness of plastic relative to purely fixed traits because organisms can better match their phenotype to their environment (DeWitt and Scheiner, 2004; Viney and Diaz, 2012). However, not all plasticity is adaptive and elucidating potentially adaptive benefits of phenotypically plastic traits requires an understanding of how plasticity differs among genotypes and how it is expressed in diverse environments.

Phenotypic plasticity is rife among nematodes (Viney and Diaz, 2012). For example, in free-living nematodes, such as *Caenorhabditis elegans* and *Pristionchus pacificus*, development can follow two alternative pathways: direct or indirect, with the latter involving an inactive dauer larval stage. The switch between these pathways represents a facultative developmental choice driven by environmental conditions, such as food availability, population density and temperature (Lee, 2002; Hu, 2007; Brown et al., 2011; Sommer and Ogawa, 2011). Another described example of phenotypic plasticity is seen in the mouth morphology of *P. pacificus* and other diplogastrid nematodes, with differing mouth forms representing an irreversible development choice that can be controlled by starvation in early larval stages (Bento et al., 2010; Ragsdale et al., 2013).

While the molecular underpinnings of some developmental processes associated with phenotypic plasticity have been carefully elucidated in the last decades, ecologically-relevant phenotypic variation remains poorly understood for most nematode species (Cutter et al., 2010). Further, the by now well-known examples of phenotypic plasticity described above represent environmentally-driven genotypic switches that lead to fixed phenotypes (i.e. polyphenisms), whereas much less detail is known about the expression of plasticity stemming from quantitatively variable traits. This is undoubtedly because the molecular mechanisms underlying continuously variable phenotypes are harder to decipher. However, several *Caenorhabditis* species have been shown to have varied adult body and brood sizes depending on larval food intake (Viney and Diaz, 2012). Different genotypes of the hermaphroditic *C. elegans* have quantitatively different responses to dauer pheromones, food concentration signals (Viney et al., 2003; Harvey et al., 2008), and bacteria (Schulenburg and Müller, 2004), as well as showing natural variation in traits, such as copulatory plug formation (Hodgkin and Doniach, 1997) and out-crossing (Teotónio et al., 2006). Likewise, *P. pacificus*, also hermaphroditic, presents phenotypic variation in degrees of dauer formation (Mayer and Sommer, 2011), chemoattraction (McGaughran et al., 2013), and out-crossing (Click et al., 2009). In general, hermaphroditic species represent a unique advantage for the analysis of phenotypic variation within and among populations because independent isogenic lines, each absent genetic variation, can be cultured and compared.

As ectotherms living at the same temperature as their surroundings, nematodes can be expected to exhibit large plasticity in their physiology, particularly in environments subject to temperature fluctuation (Hawes and Bale, 2007). In particular, low temperatures present several problems for nematodes, ranging from difficulties in molecular transport to the mechanical damage of cells with freezing (Wharton, 2011). Nematodes have evolved several strategies to cope with extremely cold temperatures, including freeze avoidance, freeze tolerance and cryoprotective dehydration (Lee, 1991; Convey and Worland, 2000a; Wharton, 2003; Wharton, 2011). In fact, the Antarctic nematode *Panagrolaimus davidi* is a model for cold tolerance (Wharton, 2002; Wharton, 2003), and it has been shown to survive temperatures as low as −80°C (Wharton and Brown, 1991). Comparatively fewer data are available on the cold tolerance of other nematode species (e.g. Smith et al., 2008); particularly those surviving sudden cold exposures in otherwise relatively warm environments, while studies targeting intraspecific variation and phenotypic plasticity of cold tolerance remain disproportionately scarce. As ubiquitous and ecologically-relevant species, nematodes represent a model for how other soil organisms might or might not respond to changing environmental conditions. In the face of predicted climate change scenarios, limitations in cold tolerance knowledge are therefore particularly unfortunate because they mean we lack understanding about the potential ability of nematodes to withstand unpredictable environments.

**Fig. 1.**
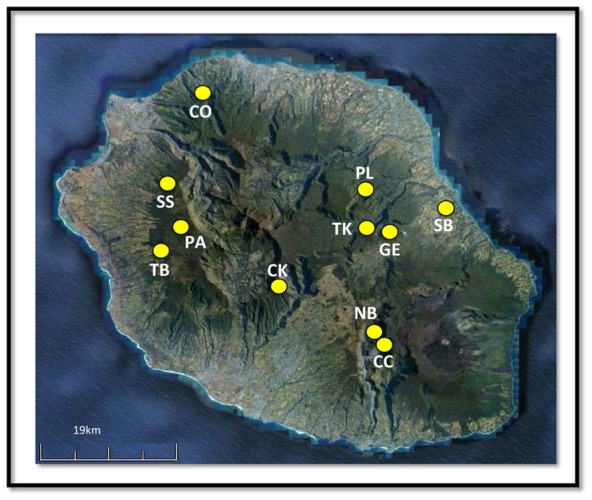
La Réunion sampling locations. Map of La Réunion Island with sampling location codes referred to in the text. See supplementary material Table S1 for further sample information.

Here, we investigate how *P. pacificus* samples collected from a variety of widespread environments may respond differently to low temperature exposure. We contrast responses among different types of cold exposure (rapid or gradual), and we examine whether geographic location among a subset of samples from La Réunion Island (Fig. 1) is an important determinant of cold tolerance variation. Importantly, for species in which genetic clones are available, different individuals of the same genotype can be placed in different environments to determine environment-dependent effects independent of genotype. Thus, we consider cold tolerance under different environmental culturing conditions to determine its sensitivity in *P. pacificus* in a genotype-by-environment (G × E) framework.

## MATERIALS AND METHODS

### Animal collection and rearing

Isogenic nematode lines without microbial or fungal contamination were thawed from Sommer Laboratory frozen stock for phenotypic analysis. Each line was reared through at least five generations (>3 weeks) at the standard laboratory culture temperature of 20°C before phenotyping, with cultures maintained on NGM agar plates seeded with the *Escherichia coli* OP50 strain. A total of 188 samples (representing independent hermaphroditic lines) were chosen to span a range of geographic locations encompassing the laboratory's collection of *P. pacificus* from tropical and temperate locations across Europe, Asia, South Africa, and America (supplementary material Table S1). A subset of these (n = 131) were selected to cover eleven geographic locations on La Réunion Island (Mascarene Islands, Indian Ocean; Fig. 1) to provide a case study allowing examination of the effects of geography on cold tolerance among samples (supplementary material Table S1). Previous work has shown that geographic location on La Réunion Island is highly correlated with site-averaged environmental data including minimum temperature and precipitation (McGaughran et al., 2014), thus ‘location’ serves as a proxy for environmental factors in the current study.

### Cold tolerance assays

For each sample, nematodes were washed off NGM agar plates into Eppendorf tubes containing a balanced salt solution (BSS; composition of 50 mOsmKg^−1^, using anhydrous components in 1 L distilled water: 417 mg NaCl, 64 mg KCl, 328 mg MgCl_2_, 569 mg Ca(NO_3_)_2_, 701 mg CaSO_4_, 208 mg MgSO_4_) to ensure osmotic stability (Wharton, 2010). After low-speed centrifugation to pellet the samples, the volume of BSS was reduced to 50 µl to yield a concentrated suspension containing approximately 100 adult nematodes per sample. This suspension was then added to the well of a PCR plate, which was transferred to a metal rack and placed into the fluid (absolute ethanol; Sigma–Aldrich Chemie GmbH; Munich, Germany) of a refrigerated circulator (Glacier G50 Bath with AC200 controller; Thermo Fisher Scientific GmbH, Schwerte, Germany).

Two assays were performed for each sample: (1) Direct exposure (DE); and (2) Gradual exposure (GE). In DE assays, samples were directly exposed from their culture temperature of 20°C to −5°C for a period of 1 h. In GE assays, nematodes were gradually exposed to the test temperature using a gradient approach – temperature was ramped from 4°C to −5°C at a cooling rate of 0.5°C.min^−1^, held at −5°C for 30 min and ramped back up to 4°C at 0.5°C.min^−1^. In both assays, following temperature treatment, samples were transferred from the assay plate to a new OP50-seeded NGM plate and scored for percent survival at 24 h. An additional assay was performed to test short-term acclimation effects. In this assay, samples that had been maintained at 20°C were transferred to 4°C incubators for a 7-day period before both the DE and GE assays were performed. During these 7 days, samples did not proceed through an additional generation hence, the assay tests cold tolerance of a given sample following its exposure to 4°C treatment. Finally, to assess genotype-by-environment effects, a subset of more densely-collected (supplementary material Table S1) La Réunion samples were cultured at two additional temperatures (15°C and 25°C), and assayed as above.

To seed the rapid freezing of samples during all cold treatment assays, a small ice crystal was added to each well of the 96-well plate once it had reached −5°C. In all cases, a control was used, which consisted of samples washed into BSS and treated exactly the same as test samples, but staying at 20°C during the course of the assay instead of undergoing a cold treatment; these controls always resulted in >99% nematode survival per sample. The freezing survival temperature of −5°C was chosen because it is the lowest recorded air temperature on La Réunion Island (Jumaux et al., 2011). The conditions of the DE and GE assays were determined in a series of range-finding experiments performed at the beginning of the experiment. Six replicates of each assay were performed.

### Statistical analysis

All statistical analyses were performed in the program MINITAB ver. 14 (Minitab Inc., Pennsylvania, United States). Examination of the raw data provided an indication that the distribution of sample responses was not normal, and this was confirmed with Anderson–Darling tests (AD>3.0; *P*<0.005). The departure was due to the presence of three distinct classes of response among samples, each with non-overlapping means and variability (see Results). Hence, the data were grouped into these three (normally distributed) classes and each class was analysed separately in all statistical tests.

One-way ANOVAs were used to confirm significant differentiation between the three phenotypic classes and to determine whether there were any differences in mean survival of samples among assays (DE or GE). A one-way ANOVA was also used to assess whether pre-exposure within one developmental generation to a cold temperature (4°C) significantly increased mean survival among acclimated samples. Differences among genotypes (i.e. hermaphroditic samples) were analysed using our more densely-collected Réunion Island dataset in the context of genotype-by-environment (G × E) effects by assessing whether samples had different mean phenotypic responses to cold treatment after culture at different environmental temperatures. Specifically, ANOVA (GLM) was used, with genotype (i.e. sample), environment (i.e. culture temperature), and their interaction, assessed. Finally, we analysed the Réunion data to see whether mean cold tolerance varied by geographic location using one-way ANOVA. Bonferroni correction was used following multiple-hypothesis testing where relevant.

## RESULTS

### Three phenotypic classes of cold tolerance among samples

**Fig. 2.**
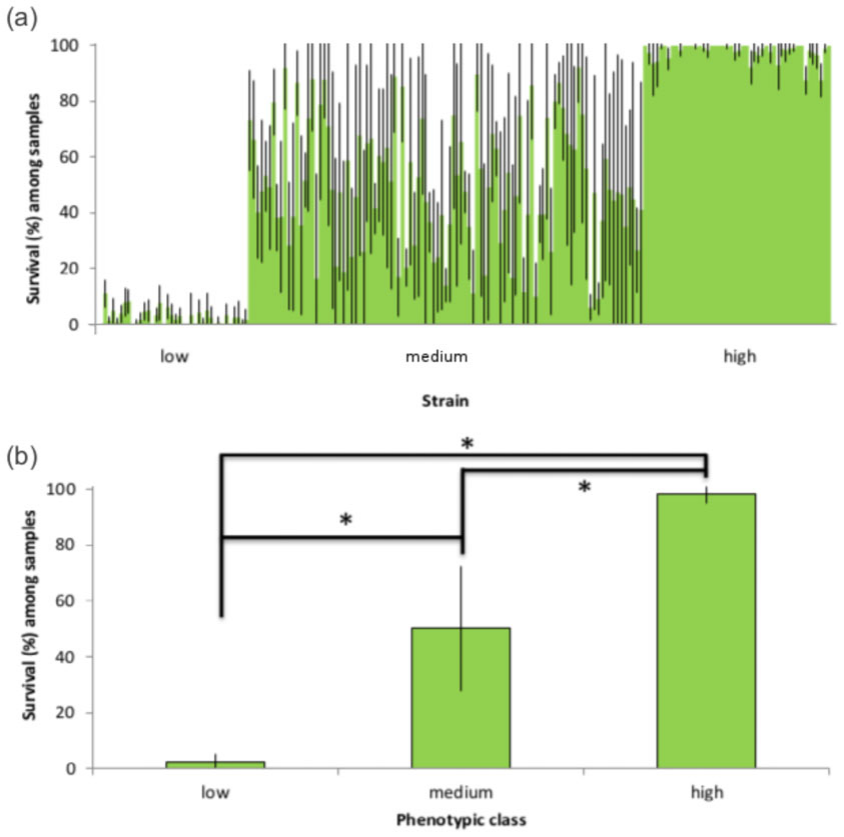
Three phenotypic classes of cold tolerance among *Pristionchus pacificus* samples. Mean percentage survival among samples of *P. pacificus* in direct cold exposure assays. (a) Each bar represents the mean of six replicate assays per sample, with error bars corresponding to standard deviation and the grouping of samples into three phenotypic classes (‘low’  =  non-cold tolerant; ‘medium’  =  cold tolerant variable; ‘high’  =  cold tolerant), as seen on the *x*-axis. (b) Mean survival (%) among samples is shown, grouped according to the three phenotypic classes. *Error bars correspond to standard deviation and significance.

A total of 188 samples representing independent hermaphroditic lines of *P. pacificus* from tropical and temperate locations across Europe, Asia, South Africa, and America (supplementary material Table S1) were analysed for their mean cold tolerance in a series of assays. Survival of exposure to low temperatures was characterized by a high degree of natural variation in *P. pacificus* (Fig. 2a). Specifically, phenotypic responses among samples fell into three classes, which had non-overlapping variability and were significantly different (F_2,466_ = 358.53; *P*<0.001) (Fig. 2b; supplementary material Table S1). These three classes correspond to ‘low’, ‘medium’, and ‘high’ mean survival, where samples are strictly non-cold tolerant (n = 39, <20% survival, <10% standard deviation, s.d.), highly variable/plastic (n = 101, 20–80% survival, ∼20% s.d.), and cold tolerant (n = 48, >80% survival across replicates, and <10% s.d.), respectively (Fig. 2b; supplementary material Table S1).

### Variation in cold tolerance with cooling rate and acclimation

**Fig. 3.**
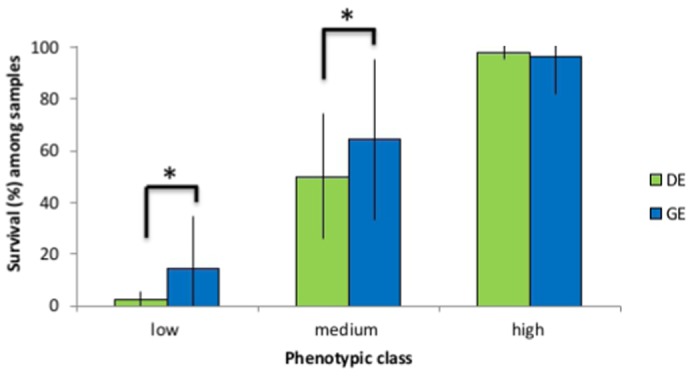
Variation in cold tolerance with cooling rate. Bar plot of mean survival responses of *Pristionchus pacificus* samples to cold tolerance, showing significantly increased survival of samples in the gradual exposure (GE) vs. the direct exposure (DE) assays for each phenotypic class. *Significance.

ANOVA was used to determine whether there were any significant differences in mean survival among samples exposed to low temperature in direct (DE) or gradual (GE) assays. We found a significant difference in mean survival for both ‘low’ and ‘medium’ phenotypic classes (F_1,76_ = 13.34; *P*<0.001 and F_1,200_ = 13.79; *P*<0.001, respectively), but not for the ‘high’ phenotypic class (Fig. 3; Table 1). Specifically, more samples survived better in their exposure to the GE assay than the DE assay for the ‘low’ and ‘medium’ phenotypic classes (Fig. 3; Table 1).

**Table 1.**
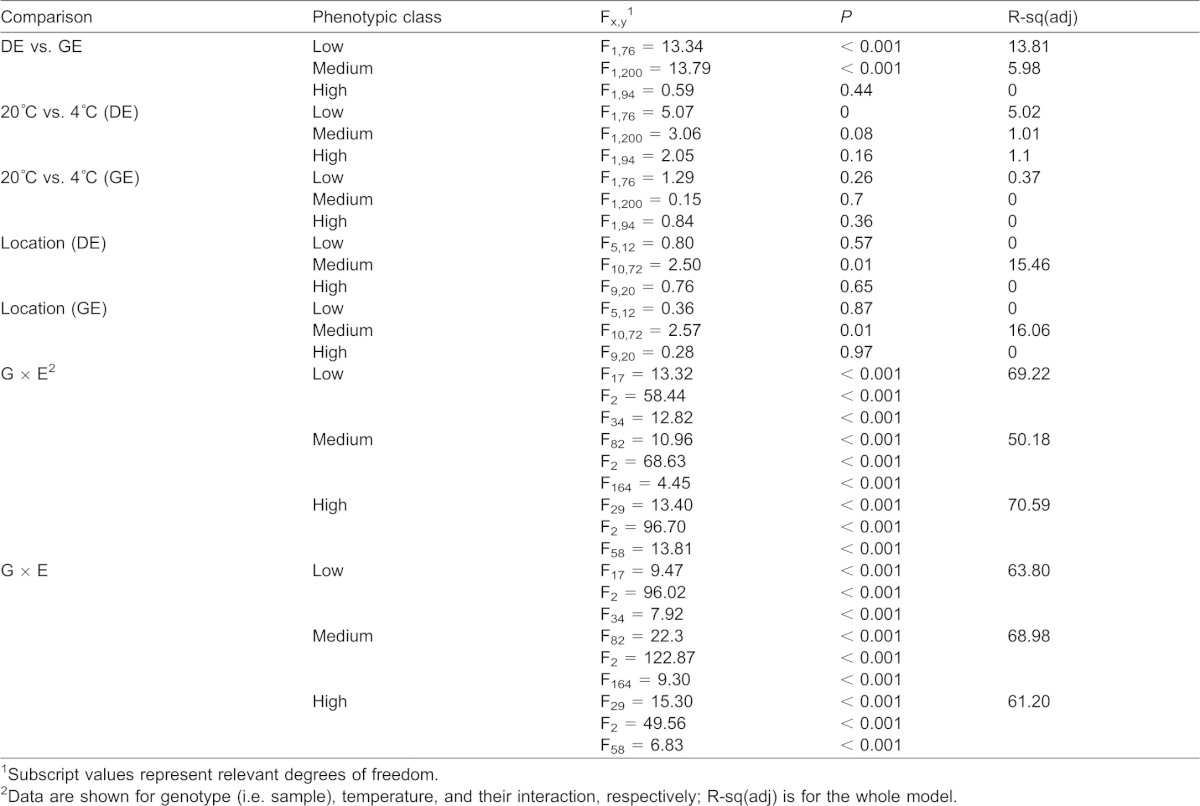
Results of various ANOVA tests for significant differentiation in mean cold tolerance among assay type (DE vs. GE), acclimation (20°C vs. 4°C), and location

**Fig. 4.**
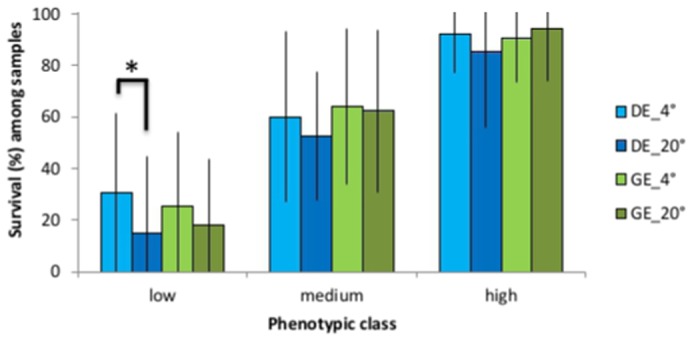
Variation in cold tolerance with acclimation. Bar plot of mean survival responses of *Pristionchus pacificus* samples to gradual exposure (GE) and direct exposure (DE) cold tolerance assays, both with and without a 4°C acclimation pre-treatment. *Acclimation significantly increased survival of samples in the DE assay.

To compare survival proportions in DE and GE assays among samples that both did and did not receive a 4°C pre-exposure treatment, ANOVA was used. We identified a significant increase in mean survival for samples in the ‘low’ phenotypic class for the DE assay (F_1,76_ = 5.07; *P* = 0.003) (Fig. 4; Table 1).

### Cold tolerance is subject to significant G × E effects

ANOVA (GLM) was used to examine G × E effects among La Réunion samples (n = 131; Fig. 1) that had been cultured at three temperatures (15°C, 20°C, 25°C), and assayed for cold tolerance in DE and GE assays. In all three phenotypic classes and for both DE and GE assays, genotype, temperature, and their interaction all had highly significant (*P*<0.001; Table 1) effects on cold tolerance.

### Cold tolerant classes are widespread across lineages and geographic locations

We next wanted to determine whether mean cold tolerance among samples varied by geographic location in our La Réunion dataset, for which samples were densely collected over eleven geographic locations that span several environmental gradients (McGaughran et al., 2014). One-way ANOVA tests found differences in mean survival among La Réunion geographic locations in the ‘medium’ phenotypic class; however, these were not significant after Bonferroni correction (Table 1).

## DISCUSSION

Within populations, individuals are rarely identical; rather, there often exists a pronounced degree of variability, independent of age or body size, which natural selection can act upon to optimise the fitness of individuals (Morozov et al., 2013). At the same time, most diploid organisms with sexual reproduction show high degrees of genetic variation often among individuals of the same population, which can contribute to observed phenotypic variability in any given trait. Organisms propagating as self-fertilizing hermaphrodites overcome these limitations because they produce isogenic (nearly homozygous) female lines. We show here that individuals of the nematode, *P. pacificus*, exhibit high levels of natural variation in their ability to survive cold exposure, corresponding to three distinct phenotypic classes: surviving cold treatment very well (>80% mean survival), very poorly (<20%), or very variably (20–80%).

In our cold tolerance assays, mean survival of gradual exposure to low temperatures was higher than mean survival of direct exposure. Further, acclimation to a low temperature treatment enhanced later mean tolerance of direct cold exposure among ‘non-cold tolerant’ samples (i.e. those in the ‘low’ phenotypic class according to the direct exposure assay without acclimation). Collectively, these results indicate that cooling rate and acclimation temperature have significant main effects on mean cold tolerance. This conforms well with cold tolerance literature for invertebrates, where an increased level of survival in the face of slower temperature cooling rates may indicate that animals are able to synthesise protective compounds (e.g. ice active proteins or cryoprotective compounds) given more time (Sinclair, 2001; Wharton, 2011). Meanwhile, acclimation treatments have been shown to improve cold tolerance in insect species (e.g. Mabbett and Wharton, 1986; Forge and MacGuidwin, 1990; Forge and MacGuidwin, 1992; Behm, 1997; Jagdale and Gordon, 1998; Smith et al., 2008), by inducing changes in gene expression (e.g. Purać et al., 2008; Burns et al., 2010). In the Antarctic nematode, *Plectus murrayi*, pre-exposure to slow dehydration improves not just extreme desiccation survival, but also promotes enhanced cold and freeze tolerance (Adhikari et al., 2010).

The presence of three distinct phenotypic responses among samples in our assays suggests that some individuals may be less set to cope with sudden cold temperature events than others. Our G × E analysis supported this, indicating a pattern of different mean phenotypic responses among genotypes to different environments. The distribution of mean phenotypic response among individuals on La Réunion Island was geographically widespread. For example, samples with low mean survival were collected from six of the eleven Réunion collection locations, while those with high mean survival were originally collected from ten of these locations. Samples falling into the ‘medium’ phenotypic class (variably cold tolerant) were found in all Réunion locations.

Variable plasticity among populations is not uncommon in the literature – sometimes referred to as ‘bet-hedging’, this strategy produces a phenotypically heterogeneous set of individuals, each of which may develop into several specialised types (DeWitt and Scheiner, 2004; Ragsdale et al., 2013). A well characterised example of this exists in the Antarctic cold tolerance literature, where populations of the springtail *Cryptopygus antarcticus* have been shown to exhibit bimodal super-cooling point (SCP – the temperature at which an insect freezes) distributions; although most individuals have relatively high freezing points (−5°C to −7°C), a proportion have much lower freezing points (−18°C to −25°C), even in summer (Rothery and Block, 1992; Convey and Worland, 2000b; Worland and Convey, 2001). This plasticity has been suggested to provide insurance against unpredictable freeze events during the polar summer (Klok and Chown, 1998), although it may also be related to the presence of food in the gut rather than to phenotypic differences among individuals (but see Worland and Convey, 2001). In tropical La Réunion Island, the need for insurance against snap cold events may seem dubious. However, among high altitude sites (CC, CK, NB), the lowest recorded minimum temperature was −5°C, while locations at ∼1500 m a.s.l. (SS, PA, TB) can reach minimum temperatures of −1.7°C, and even the lowest altitude sites still approach 10°C at times (Jumaux et al., 2011). The region is also subject to unpredictable and harsh weather systems (e.g. tropical cyclones) on a regular basis (Jumaux et al., 2011).

Phenotypic diversity has been described as an adaptive response to unpredictable environments (e.g. Donaldson-Matasci et al., 2010), and genetic variation in cold tolerance has been reported in natural systems contrasting tropical/temperate zones and high/low altitudes (e.g. Hoffmann et al., 2002; David et al., 2003; Ayrinhac et al., 2004; Castañeda et al., 2005). Such geographic variation in fitness-related traits suggests that these patterns may have been shaped by natural selection. Several nematode species have also been shown to respond to artificial selection on cold tolerance, supporting genetic adaptation to temperature stress resistance in the laboratory (e.g. Grewal et al., 1996; Jagdale and Gordon, 1998). On the other hand, the nematode *Panagrellus redivivus*, which associates with tree and plant resin in its natural habitat and is exposed to low temperatures in temperate regions during winter, exhibits only modest cold tolerance (Hayashi and Wharton, 2011). However, high cold tolerance need not be a currently adaptive trait to be maintained in nematode populations. Indeed, while the Antarctic nematode *P. davidi* has much higher tolerance to cold exposure than nematodes from temperate environments, a study by Smith et al. showed that temperate species still show some proportion of survival to exposure of −3°C for 30 minutes (Smith et al., 2008). Thus, cold tolerance plasticity may be a by-product of evolutionary history. In the case of *P. pacificus*, ‘pre-adaptation’ to cold exposure may be expected if, for example, this species previously colonised high altitude mountain habitat in other regions of its global ancestral distribution – or in fact it may relate more generally to functional changes related to developmental temperature. A body of largely Antarctic-focused research has also suggested, based on similarity at the cellular level in responses of nematodes and insects to cold tolerance and desiccation stress, that many of the physiological and molecular responses to cold may have originally been adaptations for desiccation stress (e.g. Ring and Danks, 1994; Block, 1996; Danks, 2000). Finally, migration among *P. pacificus* populations may also account for the presence of diversely cold tolerant individuals at some locations; however, genetic analyses have shown that populations of *P. pacificus* on La Réunion Island are relatively isolated in terms of gene flow (Morgan et al., 2012; McGaughran et al., 2014).

In summary, *P. pacificus* individuals were diversely capable of surviving low temperature exposure. Phenotypic plasticity and G × E among samples and populations may represent bet-hedging survival strategies that allow some individuals to maximise their fitness in different environments. Collectively, this work adds data from tropical environments to the cold tolerance literature for invertebrates, shedding light on the evolution of this important life history trait and the divergent ways in which nematodes may withstand the stresses imposed by their local environments.

## Supplementary Material

Supplementary Material
