# Supplementary material for: Natural variation in cold tolerance in the nematode Pristionchus pacificus: the role of genotype and environment
Source: Biol Open. 2014 Aug 22;3(9):832–8. doi: 10.1242/bio.20148888 (PMC4163660; doi:10.1242/bio.20148888)
Supplement: Supplementary Material [file supp_3_9_832__index.html]

Natural variation in cold tolerance in the nematode Pristionchus pacificus: the role of genotype and environment — Supplementary Material 

# Natural variation in cold tolerance in the nematode *Pristionchus pacificus*: the role of genotype and environment

## bio.20148888 Supplementary Material

**Files in this Data Supplement:**

- Table S1 - *Pristionchus pacificus* sampling information.
