# Supplementary material for: Natural variation in cold tolerance in the nematode Pristionchus pacificus: the role of genotype and environment
Source: Biol Open. 2014 Aug 22;3(9):832–8. doi: 10.1242/bio.20148888 (PMC4163660; doi:10.1242/bio.20148888)
Supplement: Supplementary Material [file supp_bio.20148888_Table_S1.docx]

**Table S1. *Pristionchus pacificus* sampling information.** (a) Sampling information summary for the 188 *Pristionchus pacificus* strains used in this study, including the number of samples from each location and, for La Réunion strains, the collection location. (b) Additional sample information, including sample code, mean survival of DE and GE assays (± s.d.), subsequent categorisation into one of three phenotypic classes (‘low’, ‘medium’, or ‘high’ survival in cold tolerance assays), and location and population information.

(a)

**Location Population No. N(total)**

Bali 1

Bolivia 7

California 3

China 3

Costa Rica 2

Hawaii 1

India 1

Japan 12

La Réunion 131

*Le Cratère Commerson (CC)* 10

*Coteau Kerveguen (CK)* 10

*Colorado(CO)* 10

*Grand Etang (GE)* 10

*Nez de Bœuf (NB)* 7

*Palmistes (PA)* 6

*Plaines des Lianes (PL)* 10

*Saint Benoit (SB)* 22

*San Souci (SS)* 10

*Trois Bassin (TB)* 30

*Takamaka (TK)* 6

Mauritius 18

Madagascar 1

Montenegro 1

Ohio 1

Poland 1

South Africa 2

Switzerland 1

Turkey 1

Washington 1

(b)

**Sample Mean survival (DE) Mean survival (GE) Category Location Population**

RS2333 4.83(4.76) 0.56(1.36) low California

RS5296B 4.58(3.32) 2.22(2.72) low California

RS5297B 5.00(4.08) 1.67(1.83) low California

CostaRica7 0.00(0.00) 0.00(0.00) low Costa Rica

JU138B 0.00(0.00) 12.08(16.00) low Hawaii

RS5200B 8.33(4.59) 25.28(24.93) low India

JU482B 11.11(5.02) 55.67(41.58) low Japan

RS5188B 3.89(3.28) 58.33(41.60) low Japan

RS5195B 7.92(5.10) 36.17(26.72) low Japan

RS5208 0.00(0.00) 13.19(4.61) low Japan

RSC172 1.67(4.08) 0.00(0.00) low La Réunion *Coteau Kerveguen (CK)*

RSC002 0.00(0.00) 0.00(0.00) low La Réunion *Le Cratère Commerson (CC)*

RSC005 3.33(4.22) 20.00(39.55) low La Réunion *Le Cratère Commerson (CC)*

RSC046 0.00(0.00) 3.33(5.16) low La Réunion *Palmistes (PA)*

RSC049 2.50(4.18) 56.67(33.12) low La Réunion *Palmistes (PA)*

RSC050 2.50(6.12) 0.56(1.36) low La Réunion *Palmistes (PA)*

RS5415 3.33(2.58) 8.89(7.79) low La Réunion *Saint Benoit (SB)*

RS5418 7.78(6.55) 3.33(4.08) low La Réunion *Saint Benoit (SB)*

RS5423 0.00(0.00) 15.00(12.25) low La Réunion *Saint Benoit (SB)*

RSB069 0.00(0.00) 3.33(8.16) low La Réunion *Saint Benoit (SB)*

RSB070 4.17(4.92) 0.00(0.00) low La Réunion *Saint Benoit (SB)*

RSB071 0.67(1.63) 0.00(0.00) low La Réunion *Saint Benoit (SB)*

RSB072 5.00(6.32) 2.22(2.72) low La Réunion *Saint Benoit (SB)*

RSB073 2.50(4.18) 65.00(50.50) low La Réunion *Saint Benoit (SB)*

RSB079 0.00(0.00) 0.00(0.00) low La Réunion *Saint Benoit (SB)*

RSB080 0.83(2.04) 0.00(0.00) low La Réunion *Saint Benoit (SB)*

RSA096 6.33(4.63) 30.00(15.41) low La Réunion *San Souci (SS)*

RS5402 0.00(0.00) 0.00(0.00) low La Réunion *Trois Bassin (TB)*

RSA616 3.33(4.08) 3.89(2.51) low Mauritius

RSA621 1.67(2.04) 0.00(0.00) low Mauritius

RSA622 3.00(3.29) 0.33(0.82) low Mauritius

RSA626 0.00(0.00) 50.42(41.30) low Mauritius

RSA627 0.00(0.00) 48.33(37.77) low Mauritius

RSA635 3.33(8.16) 31.67(49.16) low Mauritius

RSC170 0.56(1.36) 0.00(0.00) low Mauritius

RS5134B 0.83(1.29) 1.67(1.51) low Ohio

RS5295B 1.67(2.79) 18.56(36.18) low Switzerland

RS5282 0.56(1.36) 2.22(1.72) low Turkey

PS1843 1.33(1.63) 0.56(1.36) low Washington

RS5160B 66.25(21.32) 94.86(6.84) medium Japan

RS5194B 40.28(16.55) 62.00(46.96) medium Japan

RS5197 47.81(25.77) 98.67(2.07) medium Japan

RS5210-2 53.33(12.65) 80.42(16.99) medium Japan

RS5215-3 49.17(22.45) 95.33(7.87) medium Japan

RS5217B 79.67(11.96) 98.33(1.97) medium Japan

RS5221 38.38(11.94) 76.39(22.39) medium Japan

RSB020 89.72(13.68) 85.00(32.09) medium La Réunion *Colorado(CO)*

RSC015 64.58(50.26) 87.67(20.80) medium La Réunion *Colorado(CO)*

RSC017 62.67(29.92) 90.67(12.75) medium La Réunion *Colorado(CO)*

RSC019 92.22(12.94) 90.83(20.04) medium La Réunion *Colorado(CO)*

RSC021 75.28(38.91) 90.92(16.68) medium La Réunion *Colorado(CO)*

RSC007 74.17(36.77) 72.94(34.04) medium La Réunion *Coteau Kerveguen (CK)*

RSC008 26.11(22.65) 55.56(25.88) medium La Réunion *Coteau Kerveguen (CK)*

RSC009 80.00(9.98) 73.00(8.63) medium La Réunion *Coteau Kerveguen (CK)*

RSC010 86.67(7.60) 91.67(9.83) medium La Réunion *Coteau Kerveguen (CK)*

RSC011 77.78(18.85) 96.67(4.22) medium La Réunion *Coteau Kerveguen (CK)*

RSC013 68.33(40.21) 70.22(39.74) medium La Réunion *Coteau Kerveguen (CK)*

RSC100 26.67(15.20) 46.39(35.41) medium La Réunion *Coteau Kerveguen (CK)*

RS5407 78.89(34.23) 92.83(7.98) medium La Réunion *Grand Etang (GE)*

RS5408 87.78(13.80) 93.75(15.31) medium La Réunion *Grand Etang (GE)*

RS5410 71.11(35.88) 71.67(35.45) medium La Réunion *Grand Etang (GE)*

RSA046 67.78(43.08) 87.08(21.70) medium La Réunion *Grand Etang (GE)*

RSA048 26.11(36.48) 67.50(38.18) medium La Réunion *Grand Etang (GE)*

RSA049 65.00(28.11) 58.71(24.41) medium La Réunion *Grand Etang (GE)*

RSB001 47.67(7.53) 47.08(5.10) medium La Réunion *Le Cratère Commerson (CC)*

RSB005 35.00(19.06) 71.25(24.63) medium La Réunion *Le Cratère Commerson (CC)*

RSB013 11.11(15.52) 17.78(15.94) medium La Réunion *Le Cratère Commerson (CC)*

RSC001 85.83(19.28) 94.44(13.61) medium La Réunion *Le Cratère Commerson (CC)*

RSC003 10.00(12.25) 35.69(32.52) medium La Réunion *Le Cratère Commerson (CC)*

RSC004 39.44(10.20) 88.33(8.63) medium La Réunion *Le Cratère Commerson (CC)*

RSC006 39.58(16.61) 72.67(28.75) medium La Réunion *Le Cratère Commerson (CC)*

RSA076 63.33(43.20) 98.33(3.03) medium La Réunion *Nez de Bœuf (NB)*

RSB033 55.83(45.87) 80.28(34.65) medium La Réunion *Nez de Bœuf (NB)*

RSB035 17.50(40.47) 1.67(4.08) medium La Réunion *Nez de Bœuf (NB)*

RSC035 55.83(40.05) 72.22(32.77) medium La Réunion *Nez de Bœuf (NB)*

RSC048 6.11(4.91) 0.00(0.00) medium La Réunion *Palmistes (PA)*

RSB059 49.22(47.97) 93.61(9.91) medium La Réunion *Plaines des Lianes (PL)*

RSB060 68.33(24.64) 75.00(27.39) medium La Réunion *Plaines des Lianes (PL)*

RSB062 63.00(9.98) 94.44(13.61) medium La Réunion *Plaines des Lianes (PL)*

RSB064 29.17(40.05) 18.17(12.11) medium La Réunion *Plaines des Lianes (PL)*

RSC051 47.22(42.03) 0.00(0.00) medium La Réunion *Plaines des Lianes (PL)*

RSC052 9.17(5.94) 28.89(19.51) medium La Réunion *Plaines des Lianes (PL)*

RSC053 37.22(27.92) 78.33(4.08) medium La Réunion *Plaines des Lianes (PL)*

RSC054 59.44(43.99) 100.00(0.00) medium La Réunion *Plaines des Lianes (PL)*

RS5412 48.33(42.50) 90.00(19.75) medium La Réunion *Saint Benoit (SB)*

RS5416 20.83(39.04) 4.17(6.65) medium La Réunion *Saint Benoit (SB)*

RS5417 47.50(32.05) 51.11(27.78) medium La Réunion *Saint Benoit (SB)*

RS5419 18.89(39.81) 0.56(1.36) medium La Réunion *Saint Benoit (SB)*

RS5420 58.89(46.84) 64.33(31.89) medium La Réunion *Saint Benoit (SB)*

RS5421 24.17(24.58) 43.11(26.22) medium La Réunion *Saint Benoit (SB)*

RS5424 45.83(47.37) 36.67(40.46) medium La Réunion *Saint Benoit (SB)*

RSB068 41.11(33.18) 96.25(5.86) medium La Réunion *Saint Benoit (SB)*

RSB074 54.44(36.08) 11.67(11.69) medium La Réunion *Saint Benoit (SB)*

RSB077 16.67(40.82) 0.00(0.00) medium La Réunion *Saint Benoit (SB)*

RSB083 46.11(35.62) 40.00(14.14) medium La Réunion *Saint Benoit (SB)*

RSA098 16.94(14.16) 46.67(29.40) medium La Réunion *San Souci (SS)*

RSA100 85.42(20.03) 86.33(16.94) medium La Réunion *San Souci (SS)*

RSA102 20.42(6.79) 1.67(1.83) medium La Réunion *San Souci (SS)*

RSA104 58.33(37.28) 40.00(31.55) medium La Réunion *San Souci (SS)*

RSA106 28.33(19.34) 0.00(0.00) medium La Réunion *San Souci (SS)*

RSA110 52.92(43.23) 86.25(18.82) medium La Réunion *San Souci (SS)*

RSA111 73.89(27.11) 81.17(28.41) medium La Réunion *San Souci (SS)*

RSC094 44.58(46.35) 82.33(13.88) medium La Réunion *Takamaka (TK)*

RSC095 47.50(49.37) 91.67(15.14) medium La Réunion *Takamaka (TK)*

RSC096 46.67(48.26) 87.22(16.11) medium La Réunion *Takamaka (TK)*

RSC097 35.00(36.32) 60.00(46.90) medium La Réunion *Takamaka (TK)*

RSC098 49.22(27.94) 81.67(28.58) medium La Réunion *Takamaka (TK)*

RSC099 45.00(49.02) 83.00(16.96) medium La Réunion *Takamaka (TK)*

RS5334 38.75(27.24) 68.89(36.37) medium La Réunion *Trois Bassin (TB)*

RS5336 92.08(14.78) 98.33(4.08) medium La Réunion *Trois Bassin (TB)*

RS5337 28.33(23.17) 52.22(42.93) medium La Réunion *Trois Bassin (TB)*

RS5351 38.75(33.91) 38.67(41.77) medium La Réunion *Trois Bassin (TB)*

RS5385 86.67(11.64) 95.33(6.41) medium La Réunion *Trois Bassin (TB)*

RS5397 35.56(32.29) 8.33(10.33) medium La Réunion *Trois Bassin (TB)*

RS5399 51.67(9.83) 88.75(8.27) medium La Réunion *Trois Bassin (TB)*

RS5403 73.89(33.56) 92.22(6.21) medium La Réunion *Trois Bassin (TB)*

RS5404 87.92(18.87) 98.83(2.86) medium La Réunion *Trois Bassin (TB)*

RS5405 16.39(37.75) 42.78(8.54) medium La Réunion *Trois Bassin (TB)*

RSA064 66.67(41.31) 84.17(18.82) medium La Réunion *Trois Bassin (TB)*

RSA066 41.67(9.13) 63.33(36.87) medium La Réunion *Trois Bassin (TB)*

RSA071 60.56(24.08) 74.11(12.51) medium La Réunion *Trois Bassin (TB)*

RSA073 58.33(26.20) 75.42(17.43) medium La Réunion *Trois Bassin (TB)*

RSA089 51.39(38.51) 98.00(2.19) medium La Réunion *Trois Bassin (TB)*

RSA091 88.89(20.18) 31.67(34.56) medium La Réunion *Trois Bassin (TB)*

RSA113 43.89(46.11) 96.25(8.02) medium La Réunion *Trois Bassin (TB)*

RSB089 74.83(31.66) 94.44(6.55) medium La Réunion *Trois Bassin (TB)*

RSB096 11.67(12.11) 65.00(42.57) medium La Réunion *Trois Bassin (TB)*

RSB120 39.44(40.79) 95.33(10.48) medium La Réunion *Trois Bassin (TB)*

RSC093 48.33(34.30) 85.42(22.94) medium La Réunion *Trois Bassin (TB)*

JU150 73.33(18.14) 90.83(7.85) medium Madagascar

RSA623 36.67(10.75) 42.78(30.22) medium Mauritius

RSA628 22.22(26.30) 66.67(51.64) medium Mauritius

RSA629 24.00(19.80) 47.50(37.08) medium Mauritius

RSA632 39.31(34.13) 35.33(35.75) medium Mauritius

RSA633 14.00(6.07) 6.00(6.57) medium Mauritius

RSA634 35.83(27.96) 37.92(35.69) medium Mauritius

RSA636 75.00(33.76) 16.67(40.82) medium Mauritius

RSA637 53.61(40.06) 0.00(0.00) medium Mauritius

RSA638 65.56(37.10) 70.00(40.00) medium Mauritius

RSC171 41.11(46.12) 75.00(41.83) medium Mauritius

RS5302 99.33(1.63) 100.00(0.00) high Bali

RS5264B 100.00(0.00) 100.00(0.00) high Bolivia

RS5265 100.00(0.00) 100.00(0.00) high Bolivia

RS5266 98.33(2.58) 100.00(0.00) high Bolivia

RS5270B 100.00(0.00) 100.00(0.00) high Bolivia

RS5271B 100.00(0.00) 99.17(1.29) high Bolivia

RS5275 100.00(0.00) 99.11(1.44) high Bolivia

RS5278 99.58(1.02) 100.00(0.00) high Bolivia

JU723 97.50(4.18) 98.33(2.58) high China

RS5279B 100.00(0.00) 100.00(0.00) high China

RS5484 100.00(0.00) 100.00(0.00) high China

CostaRica3 100.00(0.00) 99.44(1.36) high Costa Rica

RS5212B 95.42(4.01) 98.67(3.27) high Japan

RSC014 100.00(0.00) 99.00(1.67) high La Réunion *Colorado(CO)*

RSC016 100.00(0.00) 100.00(0.00) high La Réunion *Colorado(CO)*

RSC018 87.50(5.24) 98.67(2.07) high La Réunion *Colorado(CO)*

RSC020 98.33(3.03) 100.00(0.00) high La Réunion *Colorado(CO)*

RSC022 97.50(4.18) 100.00(0.00) high La Réunion *Colorado(CO)*

RSC012 99.17(1.29) 100.00(0.00) high La Réunion *Coteau Kerveguen (CK)*

RSC173 100.00(0.00) 100.00(0.00) high La Réunion *Coteau Kerveguen (CK)*

RS5409 100.00(0.00) 99.58(1.02) high La Réunion *Grand Etang (GE)*

RS5411 100.00(0.00) 100.00(0.00) high La Réunion *Grand Etang (GE)*

RSA056 99.67(0.82) 99.17(1.29) high La Réunion *Grand Etang (GE)*

RSA059 100.00(0.00) 99.33(1.63) high La Réunion *Grand Etang (GE)*

RSB008 97.78(4.04) 97.50(5.00) high La Réunion *Le Cratère Commerson (CC)*

RSB034 100.00(0.00) 100.00(0.00) high La Réunion *Nez de Bœuf (NB)*

RSB037 92.92(8.58) 99.33(1.63) high La Réunion *Nez de Bœuf (NB)*

RSC036 96.67(5.16) 100.00(0.00) high La Réunion *Nez de Bœuf (NB)*

RSC037 87.50(6.21) 97.22(4.43) high La Réunion *Palmistes (PA)*

RSC047 98.89(1.72) 100.00(0.00) high La Réunion *Palmistes (PA)*

RSB065 98.89(2.72) 97.50(2.74) high La Réunion *Plaines des Lianes (PL)*

RSB066 98.33(4.08) 100.00(0.00) high La Réunion *Plaines des Lianes (PL)*

RS5413 100.00(0.00) 100.00(0.00) high La Réunion *Saint Benoit (SB)*

RSA097 96.67(2.98) 100.00(0.00) high La Réunion *San Souci (SS)*

RSA108 98.89(2.72) 100.00(0.00) high La Réunion *San Souci (SS)*

RS5347 98.33(3.03) 97.67(5.72) high La Réunion *Trois Bassin (TB)*

RSA062 97.92(3.32) 98.89(1.72) high La Réunion *Trois Bassin (TB)*

RSA065 98.33(2.58) 100.00(0.00) high La Réunion *Trois Bassin (TB)*

RSA069 100.00(0.00) 93.33(8.43) high La Réunion *Trois Bassin (TB)*

RSA072 100.00(0.00) 100.00(0.00) high La Réunion *Trois Bassin (TB)*

RSA090 92.22(6.21) 99.44(1.36) high La Réunion *Trois Bassin (TB)*

RSA092 98.00(4.00) 0.00(0.00) high La Réunion *Trois Bassin (TB)*

RSB088 99.17(2.04) 98.89(1.72) high La Réunion *Trois Bassin (TB)*

RSA630 100.00(0.00) 78.89(36.80) high Mauritius

RS106B 93.67(11.69) 96.00(3.35) high Poland

RS5171B 94.17(9.17) 94.44(6.55) high Montenegro

RS5202B 99.67(0.82) 95.00(5.18) high South Africa

RS5205 100.00(0.00) 96.33(4.27) high South Africa
